# Acupuncture combined with traditional Chinese medicine for knee osteoarthritis: A protocol for systematic review and meta-analysis

**DOI:** 10.1097/MD.0000000000031820

**Published:** 2022-12-16

**Authors:** Ying Wang, Qi Lu, Haoze Guo, Jiabao Sun, Xiaomin Li, Hongyi Guan, Yu Gao, Bailin Song

**Affiliations:** a Department of Acupuncture and Tuina, Changchun University of Chinese Medicine, Changchun, China; b School of Nursing Beihua University, Jilin, China.

**Keywords:** acupuncture, knee osteoarthritis, meta-analysis, protocol, systematic review, traditional Chinese medicine

## Abstract

With the aging of society, the incidence of knee osteoarthritis (KOA) is increasing year by year, which seriously affects the quality of life. Acupuncture therapy has been widely used in the treatment of osteoarthritis, but there is no complete systematic review on acupuncture combined with traditional Chinese medicine. Therefore, this study aims to clarify the efficacy and safety of acupuncture combined with traditional Chinese medicine in the treatment of KOA through systematic review and meta-analysis.

**Methods:**

A structured and systematic literature search will be conducted in the following databases up to March, 20, 2022: PubMed, Embase, Cochrane Central Register of Controlled Trials, Web of Science, China National Knowledge Infrastructure, Chinese Biomedical Literature Database, Chinese Scientific and Journal Database, Wan Fang database and 2 clinical trials register platforms: Chinese Clinical Trial Registry, ClinicalTrials.gov (www.ClinicalTrials.gov/). We will use the Review Manager 5.4 software provided by the Cochrane Collaborative Network for statistical analysis. We then assessed the quality and risk of the included studies and observed the outcome measures.

**Results:**

This meta-analysis further established the efficacy of acupuncture combined with traditional Chinese medicine in the treatment of KOA.

**Conclusion:**

This meta-analysis aims to investigate the efficacy of acupuncture combined with traditional Chinese medicine on patients with KOA and provide reliable evidence. To provide more options for clinicians and patients in the treatment of KOA.

## 1. Introduction

Knee osteoarthritis (KOA) is a chronic degenerative disease with knee movement disorders, pain, deformity and other symptoms.^[1]^ Its etiology is still unclear, and it is related to age, obesity, inflammation, trauma and genetic factors. With the improvement of living standards in China, the number of obese people has increased. The aging population is becoming more and more serious, and the incidence of KOA is increasing year by year in our country.^[2–5]^ The incidence of KOA increases with age, reaching more than 40% in people aged 70 to 74 years.^[6]^ According to statistics, the prevalence of symptomatic KOA in China is 8.1%, and females are more than males.^[7]^ Therefore, the prevention and treatment of KOA is particularly important.

At present, there are many treatment methods for KOA, including oral drug therapy, shock wave therapy, radio frequency therapy, surgical treatment, rehabilitation therapy and functional exercise. However, there are many adverse reactions of western medicine, high risk and high price of surgical treatment for elderly patients, so a safe and effective treatment is urgently needed.^[8,9]^

Acupuncture and moxibustion treatment is the important component of traditional medicine of our country. It has exact curative effect in the treatment of KOA and has been more and more used in clinical practice. Studies have shown that acupuncture therapy is superior to other therapies in treating KOA and improving pain.^[10,11]^ In recent years, with the gradual deepening of the research on the mechanism of acupuncture and moxibustion in the treatment of KOA, it has provided better theoretical guidance for clinical practice. However, there are few systematic reviews on acupuncture combined with traditional Chinese medicine in the treatment of KOA. Therefore, this paper conducted a meta-analysis on randomized clinical trail (RCT) of acupuncture combined with traditional Chinese medicine to explore its therapeutic effects and effects.

## 2. Materials and methods

This systematic review protocol was registered in the PROSPERO International Registry of Systematic Reviews (ID:CRD42022366874).The protocol of this meta-analysis will be conducted and reported in accordance with the Preferred Reporting Items for Systematic Reviews and Meta-Analysis Protocols statement guidelines.^[12]^

### 2.1. Inclusion criteria

#### 2.1..1. Types of studies.

All RCTs that stated the “randomization” phrase will be included, regardless of allocation concealment or use of blinding, and published or unpublished RCTs without language restriction. Articles of the following research types will be excluded: case series, observational studies (including cohort and casecontrol studies), and retrospective studies, qualitative studies, animal experiments, review articles. There are no restrictions on study area, race, patient age, and gender.

#### 2.1..2. Types of participants.

Participants meeting nationally and internationally recognized KOA criteria, according to the American College of Rheumatology 1995 standards^[13]^ will be included, regardless of age, gender, and case source.

#### 2.1..3. Types of interventions.

Acupuncture combined with traditional Chinese medicine is the main intervention measure. Acupuncture includes regular acupuncture, electroacupuncture, scalp acupuncture, auricular acupuncture, fire needling, intradermal needling, and catgut embedding acupuncture, warm acupuncture, needle knife, etc. Traditional Chinese medicine is a traditional Chinese medicine prescription, not limited to a specific prescription or dosage form. Moxibustion, laser acupuncture, bleeding therapy, acupoint injection, etc will be excluded. Additionally, limitations to intervention intensity, frequency, and duration were not involved.

#### 2.1..4. Types of control interventions.

The control group was treated with sham acupuncture, placebo, or conventional medicine, or no treatment.

#### 2.1..5. Outcomes.

The results include efficacy and safety evaluation. The primary outcome measures included total clinical response rate, WOMAC score, visual analogue scale score, Lysholm knee function score, etc. The secondary outcome measures included Life scale score and adverse reactions.

### 2.2. Search methods

We searched 8 electronic databases of PubMed, EMBASE, Cochrane Central Register of Controlled Trials, Web of Science, China National Knowledge Infrastructure, Chinese Biomedical Literature Database, Chinese Scientific and Journal Database, and Wan Fang database to identify literature on RCTs for KOA. The reference lists of all included studies or relevant reports of clinical trials or reviews will be screened for additional relevant articles. The WHO International Clinical Trials Registry Platform, Chinese Clinical Trial Registry and ClinicalTrials. Gov, will be searched for ongoing or have finished trials with unpublished data. Table 1 shows PubMed search strategy.

**Table 1:** Search strategy for PubMed.

No	Search terms
#1	acupuncture. ti, mesh.
#2	acupuncture therapy. ti, ab.
#3	acupoint application. ti, ab.
#4	Or#1-#3
#5	knee osteoarthritis. ti, mesh
#6	KOA. ti, ab.
#7	Or #4-#5
#8	traditional Chinese medicine.ti.ab
#9	Or #7-#8
#10	randomized controlled trial. pt.
#11	controlled clinical trial. pt.
#12	randomized. ab.
#13	Randomly. ab.
#14	trial. ab.
#15	or #10-#14
#16	Humans/not exp animals. sh
#17	#15 not #16
#18	#4 and #7 and #9 and #1

### 2.3. Data collection and analysis

#### 2.3..1. Selection of studies.

**Figure 1.**
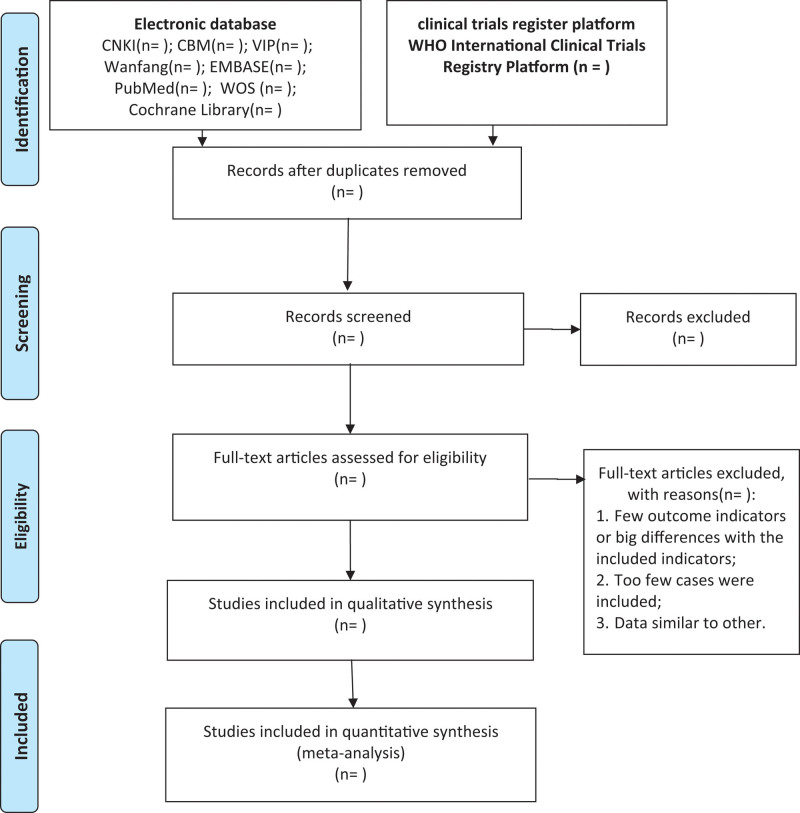
PRISMA flow diagram of study and exclusion. PRISMA = preferred reporting items for systematic reviews and meta analyses.

Clinical studies will be identified and reviewed by 2 independent reviewers (QL and JBS). In order to ensure that the reviewers have a good understanding of the background and purpose of the review, they will be trained in advance. Use NoteExpress 3.2.0 software (Available at: http://www.inoteexpress.com/aegean/) to independently manage the search results from above-mentioned databases. First, duplicate articles were deleted, and the title summaries were reviewed to further exclude the articles, and then the articles that did not meet the criteria were further reviewed. The dissenting articles will be decided after discussion by the third reviewer (YG), and the final consistency scheme will be formed. The selection process is fully elucidated in the following PRISMA flow diagram (Fig. 1).

#### 2.3..2. Data extraction and management.

Then, 2 reviewers (HZG and XML) will extract the title, first author, publication year, country, language, journal source; information of participants: gender, age, study design, sample size, intervention, type of measures, risk of bias assessment, and findings from included studies with Excel file. The results will be cross-checked by 2 examiners and any differences will be resolved by consensus through discussion with experts.

#### 2.3..3. Assessment of risk of bias and reporting of study quality.

Two independent reviewers (HYG and YG) will assess the risk of bias adopt the Cochrane risk of bias tool and complete the STRICTA checklist. And, the Jadad scale will be used to estimate the study quality.

### 2.4. Measures of treatment effect

Mean differences with 95% confidence intervals will present as the continuous data. Also risk ratio will be the expression of dichotomous data.

### 2.5. Management of missing data

If the information is missing or incomplete, we will attempt to contact the original author. If it cannot be supplemented, malformed data will be removed.

### 2.6. Assessment of heterogeneity

*I*^2^ will be used for assessing statistical heterogeneity. It is acknowledged that *I*^2^ < 25% indicates negligible heterogeneity, 25% < *I*^2^ < 50% indicates mild heterogeneity, 50% < *I*^2^ < 75% moderate heterogeneity, and *I*^2^ ≥ 75% high heterogeneity.

### 2.7. Assessment of publication bias

Over 10 studies included,^[14]^ we will take advantage of funnel plot to assess the reporting bias. Symmetrical funnel indicates no 3 publishing bias, but if the funnel is not symmetrical, which indicates publishing bias exists. *P* value will be utilized, while less than 10 studies included.

### 2.8. Data synthesis.

Quantitative analysis will be implemented using RevMan version 5.4 with 95% confidence intervals. The average change for each major and minor result will be combined. In addition, if the data is not suitable for quantitative analysis, qualitative description will be used.

### 2.9. Subgroup analysis

If possible, subgroups will be analyzed according to different acupuncture manipulation.

### 2.10. Sensitivity analysis

Robustness of the results will be assessed by sensitivity analysis performance which will focus on the processing method of missing data.

### 2.11. Grading the quality of evidence

Grading of Recommendations Assessment, Development, and Evaluation Reliability Study (GRADE) will be implemented to assess the quality of evidence. Based on the risk of bias, inconsistency, imprecision, indirection, and publication bias, GRADE grades evidence quality into 4 levels: high, medium, low, and very low.

### 2.12. Ethics and dissemination

Due to nothing of the information will be obtained from an individual participant, the systematic review does not need ethical approval.

## 3. Discussion

KOA has a high incidence in middle-aged and elderly people, and its main cause is the degeneration of articular cartilage caused by mechanical wear. Studies^[15]^ have shown that chronic low-grade inflammatory response plays an important role in the development of KOA. Clinically, symptomatic treatment is often adopted according to the patient’s condition, and surgical treatment is adopted for severe patients.^[16,17]^ Opioid painkillers, non-steroidal anti-inflammatory drugs and other drugs have certain therapeutic effects on KOA, but there will be adverse reactions in the process of medication, such as complicated gastrointestinal ulcer, arrhythmia, liver and kidney damage, etc.^[18,19]^ At present, most treatment plans, guidelines and expert consensus recommend non-drug therapy as the basis for KOA treatment.^[16,20]^

There are many kinds of treatment of traditional Chinese medicine external treatment of KOA, including traditional Chinese medicine external treatment, Chinese medicine hot iron, such as traditional Chinese medicine fumigation, acupuncture therapy, acupuncture and moxibustion as a kind of drug therapy, can directly effect on knee joint local, can have the effect of targeted in the affected area, analgesic effect is obvious, has a good effect on the improvement of the symptoms of KOA, recognized by the vast number of patients in clinical practice. Related research studies have shown that acupuncture treatment can significantly relieve pain and dysfunction and improve joint function compared with sham acupuncture and waiting treatment.^[21,22]^ The American College of Rheumatology guidelines^[17]^ state that acupuncture is recommended in cases where medical treatment does not work. In addition, the research of external treatment of KOA should be based on the inheritance and development of traditional methods of ancient Chinese medical books, combined with modern medicine to further improve the way of acupuncture and moxibustion, so as to serve the clinical practice. This systematic review will evaluate the efficacy and safety of acupuncture combined with traditional Chinese medicine in the treatment of KOA. The significance of the review and meta-analysis is to evaluate the effect in a larger sample and summarize the current findings to further provide recommendations for clinical studies, which have positive implications for the treatment of KOA.

## Author contributions

Ying Wang and Bailin Song had the original idea of this work and drafted the protocol. The search strategy was developed by all the authors and will be performed by Ying Wang, Bailin Song, Qi Lu, Haoze Guo, JiaBao Sun et al Bailin Song proposed some advice for design and revision. Qi Lu, Jiabao Sun and Yu Gao independently collected the eligible studies. Haoze Guo and Xiaomin Li completed the extraction independently. Hongyi Guan and Yu Gao assessed the bias risk and dealt with missing data. All the authors participated in this study critically revised the final version of the manuscript and confirmed the publication of this protocol.

**Conceptualization:** Ying Wang.

**Data curation:** Qi Lu, Jiabao Sun.

**Formal analysis:** Haoze Guo.

**Funding acquisition:** Bailin Song.

**Investigation:** Xiaomin Li, Hongyi Guan.

**Methodology:** Ying Wang, Qi Lu.

**Supervision:** Bailin Song.

**Validation:** Yu Gao.

**Writing – original draft:** Ying Wang.

**Writing – review & editing:** Ying Wang, Bailin Song.
